# Intranasal vaccine for Lyme disease provides protection against tick transmitted *Borrelia burgdorferi* beyond one year

**DOI:** 10.1038/s41541-023-00802-y

**Published:** 2024-02-15

**Authors:** Maria Cristina Gingerich, Nisha Nair, Jose F. Azevedo, Kamalika Samanta, Suman Kundu, Biao He, Maria Gomes-Solecki

**Affiliations:** 1 Department of Infectious Diseases, College of Veterinary Medicine, University of Georgia, Athens, GA USA; 2 CyanVac, LLC, Athens, GA USA; 3 Department of Microbiology, Immunology, and Biochemistry, University of Tennessee Health Science Center, Tennessee, USA; 4 Immuno Technologies, Inc., Memphis, TN USA; 5 Present Address: Merck & Co., West Point, PA USA

**Keywords:** Live attenuated vaccines, Bacterial infection

## Abstract

Strategies for disease control are necessary to reduce incidence of Lyme Disease (LD) including development of safe vaccines for human use. Parainfluenza virus 5 (PIV5) vector has an excellent safety record in animals and PIV5-vectored vaccines are currently under clinical development. We constructed PIV5-vectored LD vaccine candidates expressing OspA from *B. burgdorferi* (OspA_B31_) and a chimeric protein containing sequences from *B. burgdorferi* and *B. afzelii* (OspA_BPBPk_). Immunogenicity and vaccine efficacy were analyzed in C3H-HeN mice after prime-boost intranasal vaccination with live PIV5-OspA_B31_ or PIV5-OspA_BPBPk_, subcutaneous (s.c.) vaccination with rOspA_B31_+Alum, and the respective controls. Mice vaccinated intranasally with live PIV5-A_B31_ or PIV5-A_BPBPk_ had higher endpoint titers of serum antibody against OspA_B31_ at 6- and 12- months post vaccination, compared to mice vaccinated s.c. with rOspA_B31_. Neutralization activity of antibody was maintained up to 18-months post-immunization, with the response greater in live PIV5-delivered OspA vaccines, than that induced by s.c. rOspA_B31_. Challenge with infected ticks carrying 10-19 strains of *B. burgdorferi* performed at 4-, 9- or 15-months post-immunization showed increased breakthrough infections in mice vaccinated with s.c. rOspA_B31_ compared to intranasal PIV5-A_B31_ or PIV5-A_BPBPk_ at 9- and 15-months, as determined by quantification of serologic antibodies to *B. burgdorferi* proteins as well as *flaB* DNA in tissues, and by visualization of motile *B. burgdorferi* in culture of tissues under dark field microscope. These findings indicate that immunization of mice with PIV5 delivered OspA generates immune responses that produce longer-lasting protection ( > 1 year) against tick-transmitted *B. burgdorferi* than a parenteral recombinant OspA vaccine.

## Introduction

Lyme disease is a tick-borne illness caused by the spirochete *Borrelia burgdorferi* sensu lato (Bbsl), (*Borreliella genus novum* under consideration^1^). The most effective way to avoid this disease is to avoid tick-infested areas. Additional disease control strategies are necessary to reduce incidence of Lyme disease including development of safe vaccines for human use.

Outer surface protein A (OspA) is the only immunogen proven to provide 76–92% protection^2,3^ against tick-transmitted *B. burgdorferi* in fully vaccinated human subjects. Mechanistically, antibody to OspA produced upon vaccination of a host is ingested with the bloodmeal by a feeding tick and it blocks transmission of *B. burgdorferi* from the tick to the mammalian host^4–7^. The titer of anti-OspA antibody in the host’ blood must be at or above a certain level to be effective^2,8^. In the first clinical trials^2,3^, the immunization protocols required 2 shots within 1 month and a third shot 12 months after prime. After a 20-year hiatus, a re-engineered vaccine based on the C-terminus sequence of OspA from several Bbsl genospecies (VLA15)^9^ is undergoing Phase III clinical trials by Pfizer/Valneva. To maintain sufficient protective antibody levels over time, the VLA15 Phase I immunization protocol (NCT03010228) required 3 intramuscular shots at days 1, 29 and 57^10^. Other immunization protocols are being tested that require a 4^th^ shot 12 months after prime (NCT05477524). These immunization protocols further substantiate the initial finding that parenteral OspA-based vaccines do not induce prolonged immunity.

Parainfluenza virus 5 (PIV5) is a nonsegmented, negative-strand, RNA virus of the family *Paramyxoviridae*^11^. A distinctive trait of PIV5 is its ability to infect most mammalian cell types through sialic acid receptors without causing cytopathic effect. This allows for active replication of PIV5 in the respiratory tract after intranasal immunization, leading to the induction of mucosal immunity via the generation of antigen-specific IgA antibodies and long-lived IgA plasma cells, in addition to systemic humoral IgG and cell-mediated immune responses^12,13^. Live PIV5, not replication deficient, has been a component of the kennel cough vaccine administered to dogs intranasally for over 50 years. Due to potential shedding of the vaccine up to 5 days post-administration^14^, humans have been exposed to PIV5 since the vaccine came to market. However, no disease has been reported in exposed individuals, highlighting PIV5 excellent safety record. Previously, live PIV5-vectored vaccines for bacterial pathogens like *Mycobacterium tuberculosis* and *Burkholderia mallei* and *B. pseudomallei* have been tested and proven efficacious in a mouse model^15–17^. Furthermore, two PIV5-vectored vaccines for COVID-19 and respiratory syncytial virus (RSV) (CVXGA1 and BLB201, respectively) have undergone phase I clinical trials. Here, we describe the construction of a live PIV5-vectored vaccine expressing OspA from *B. burgdorferi* sensu lato. We show longer-lasting protection from challenge with *B.* *burgdorferi*-infected ticks in mice vaccinated intranasally with 2 doses of the vaccine than in mice vaccinated parenterally with 2 doses of OspA protein.

## Results

### Generation and characterization of PIV5-based vaccines expressing the outer membrane protein A (OspA)

**Fig. 1:**
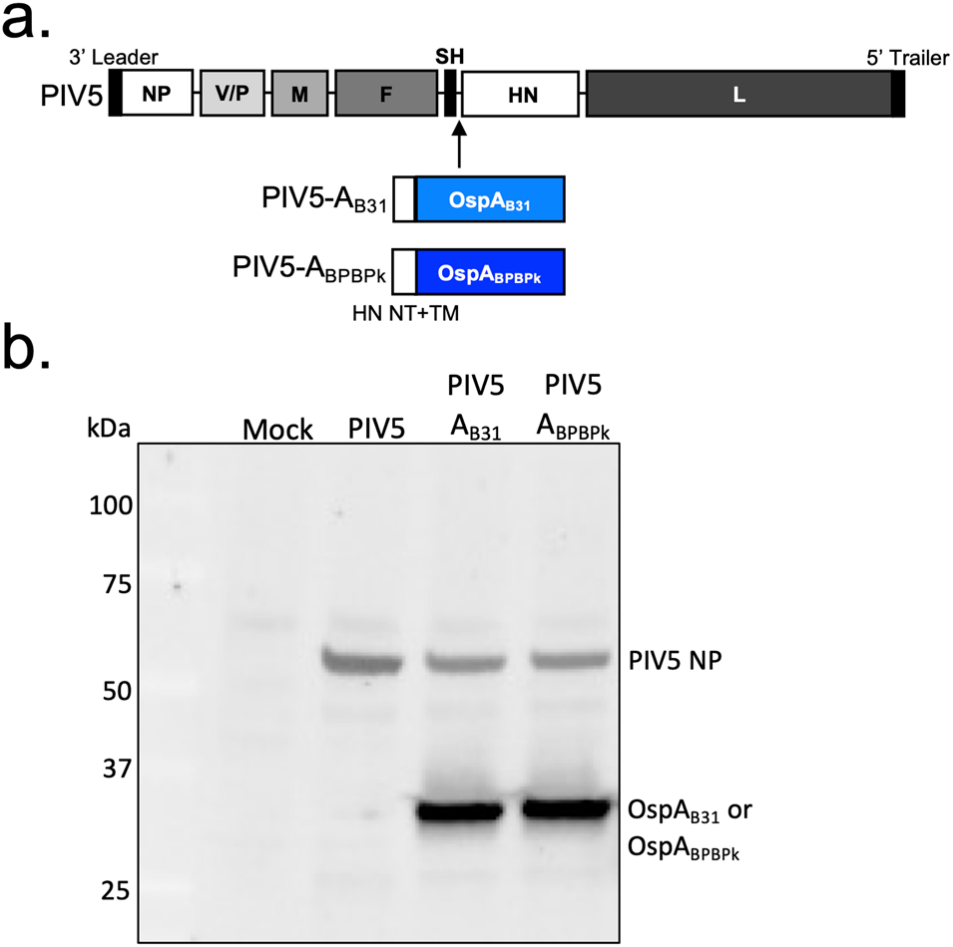
Generation and characterization of PIV5-A_B31_ and PIV5-A_BPBPk_ vaccine candidates. **a** Schematic of vaccine candidates. PIV5 has seven genes encoding for eight proteins, 3’ leader, NP, V/P, M, F, SH, HN, L, 5’ trailer. The OspA B31 and BPBPk proteins contain the N-terminus (NT) and transmembrane domain (TM) sequence from PIV5 HN protein. **b** Detection of OspA expression. Vero cells were mock-infected or infected with PIV5 vector control, PIV5-A_B31_, or PIV5-A_BPBPk_ at an MOI of 1. Forty-eight hours after infection the cells were lysed, and the lysates resolved on an SDS-PAGE gel and immunoblotted with anti-OspA (184.1) and anti-PIV5 NP (NP214mAb) monoclonal antibodies diluted 1:100. The gel and blot derived from the same experiment, and they were processed sequentially.

We generated the PIV5-A_B31_ and PIV5-A_BPBPk_ vaccine candidates by inserting the *ospA* gene sequence in the PIV5 genome between the SH and HN genes (Fig. 1a). PIV5-A_B31_ carried the full-length OspA sequence from *B. burgdorferi* strain B31. PIV5-A_BPBPk_ carried a full-length chimeric sequence of OspA in which the *B. burgdorferi* B31 amino acid sequences 165-189 and 219-273 were replaced with the respective sequences from *B. afzelii* strains (BPBPk)^18^. The N-terminus and transmembrane domain sequences from the PIV5 HN protein were also introduced directly upstream of the *ospA* gene in both vaccine candidates to improve incorporation of the bacterial protein into the PIV5 virion. The vaccine viruses were rescued as described^19^, and their genomes were confirmed through RT-PCR and sequencing. Expression of the OspA protein in PIV5-A_B31_- and PIV5-A_BPBPk_-infected cells was confirmed through Western blot assay (Fig. 1b).

### Analysis of *B. burgdorferi* strain variability in *I. scapularis* used for tick challenge

**Fig. 2:**
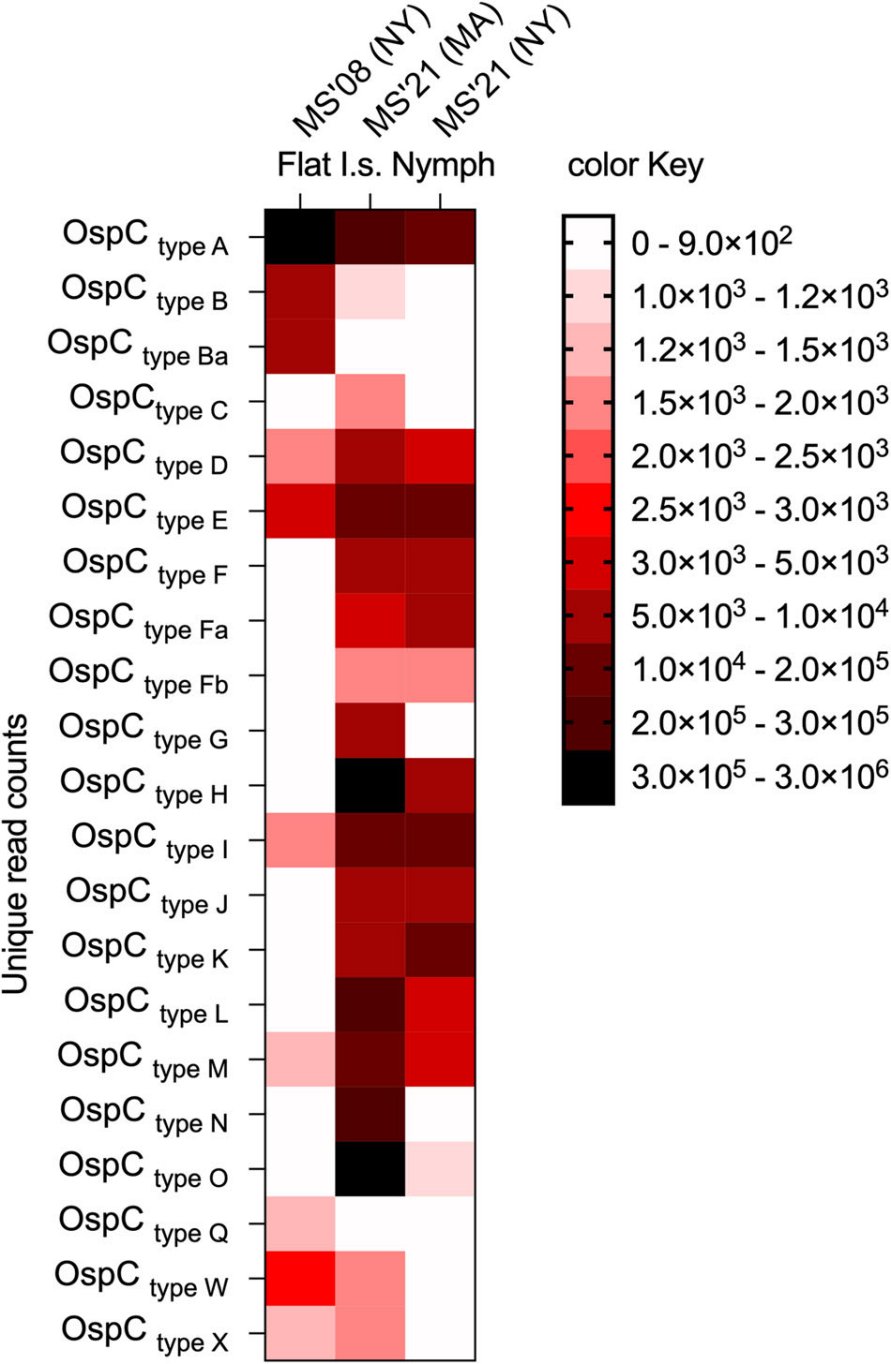
Heat map analysis of *B. burgdorferi* (*Bb*) strain variability in laboratory maintained *Ixodes scapularis* nymphal ticks. DNA purified from flat nymphal ticks was used for amplification and sequencing of the *ospC* gene by the Proton Ion Torrent Instrument. Legend: I.s., *Ixodes scapularis*; MS’08 (NY), MS’21 (MA) and MS’21 (NY) - the *Bb* culture used to produce the ticks originated from tissues from mice infected with ticks flagged in NY 2008, in MA 2021 and in NY 2021.

Sequencing OspC genes from the 3 colonies of *I. scapularis* ticks maintained in the laboratory for tick challenges (Fig. 2) showed that multi-strain MS’08/NY ticks mostly carried 10 types of OspC (A, B, Ba, D, E, I, M, Q, W, X) with >1000 unique read counts, whereas the other OspC types had <900 unique reads. MS’21/MA ticks mostly carried 19 types of OspC (A, B, C, D, E, F, Fa, Fb, G, H, I, J, K, L, M, N, O, W, X) with >1000 unique read counts, whereas the other two OspC types had <900 unique reads. MS’21/NY ticks mostly carried 13 types of OspC (A, D, E, F, Fa, Fb, H, I, J, K, L, M, O) with >1000 unique read counts, whereas the other OspC types had <900 unique reads.

### Immunization with intranasal PIV5-A_BPBPk_ or PIV5-A_B31_ induces long-lasting neutralizing antibody responses

**Fig. 3:**
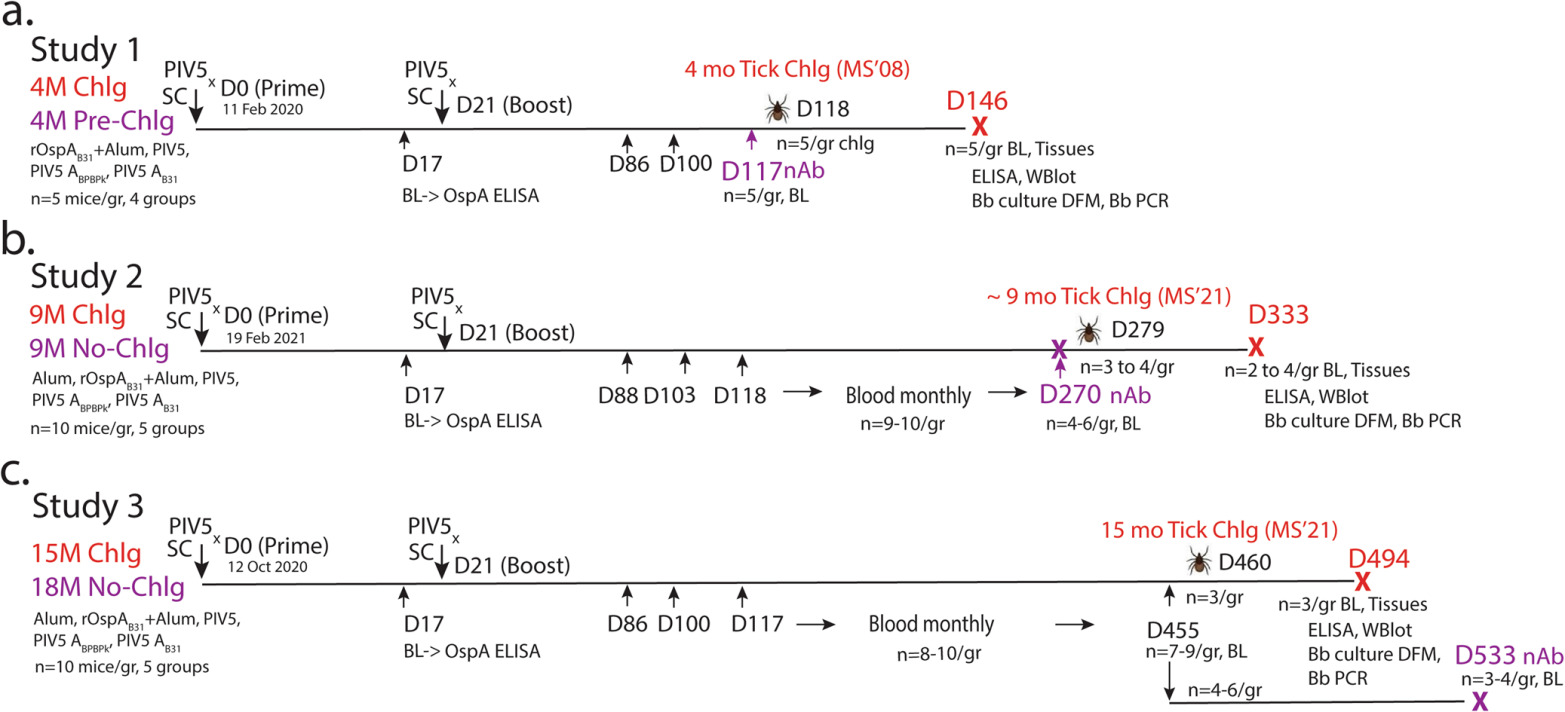
Vaccination schedule and methods for evaluation of efficacy before and after tick challenge. **a** Study 1, comprised of 4 groups of 5 mice used for neutralization assays and for tick challenge at 4-months post prime; **b** Study 2, comprised of 5 groups of 10 mice, some of which were used for neutralization assays (*n* = 4–6) and for tick challenge (*n* = 3–4) at 9-months post prime; **c** Study 3, comprised of 5 groups of 10 mice, some of which were used for tick challenge (*n* = 3) at 15-months post prime; the remaining surviving mice (*n* = 3–4) were used for neutralization assays at 18-months postprime. Legend: PIV5_x_, intranasal droplet immunization with PIV5 delivered vaccines; SC subcutaneous inoculation, M month, d day after prime, Bb *B. burgdorferi*, X euthanasia, BL blood, WBlot western blot, nAb neutralization assay, DFM dark field microscopy, MS’08 tick colony made from bladder cultures obtained from mice infected with field ticks in 2008, MS’21 tick colony made from bladder cultures obtained from mice infected with field ticks in 2021.

**Fig. 4:**
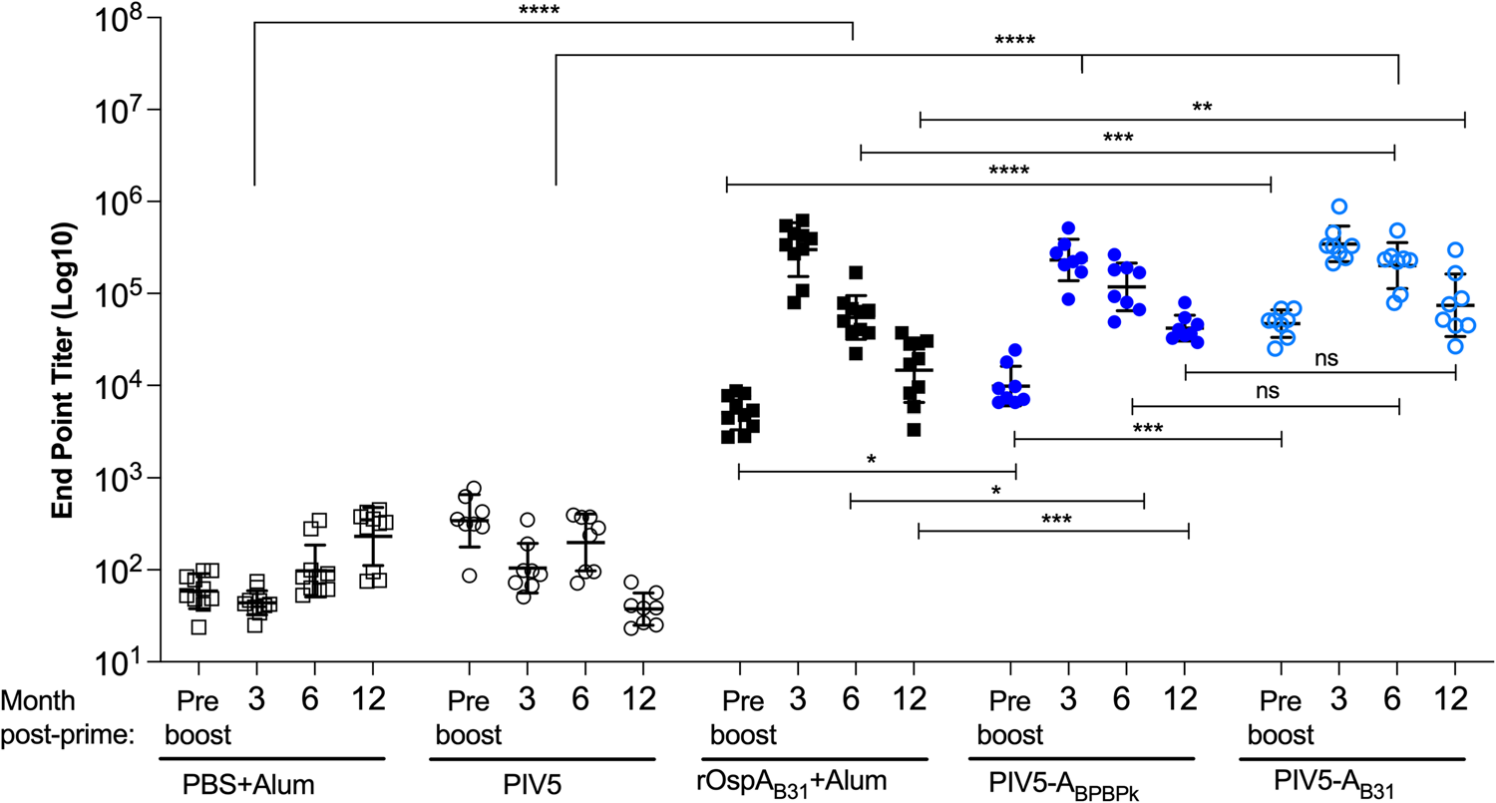
Intranasal vaccination with PIV5-A_BPBPk_ or PIV5-A_B31_ induces long-lasting IgG responses. Mice in Study 3 (15-month challenge) received two doses of alum alone or alum + 20 µg of rOspA_B31_ protein subcutaneously, or 10^6^ PFU of PIV5 vector control, PIV5-A_BPBPk_, or PIV5-A_B31_ intranasally at 21 days interval. Serum from blood collected at day 17 and months 3, 6, 12 postprime was tested against purified rOspA_B31_ and anti-OspA IgG antibody titers were quantified by ELISA. Statistical significance was calculated by Repeated Measures 2-Way ANOVA for comparisons within routes of immunization, i.e. intranasal PIV5 v PIV5-A_BPBPk_ v PIV5-A_B31_ and subcutaneous Alum v OspA_B31_+Alum; nonparametric multiple Mann-Whitney tests were used for comparisons between two OspA vaccinated groups at each timepoint. Error bars, geometric means with geometric SD. 3-month comparisons are not significant, ns, not significant, *p < 0.05, **p < 0.005, ***p < 0.0005, ****p < 0.0001.

To assess humoral IgG immune responses induced by intranasal PIV5-A_BPBPk_, intranasal PIV5-A_B31_ and s.c. rOspA_B31_ vaccines (Fig. 3), blood samples collected before tick challenge from the three studies were used for determination of anti-OspA antibody (1:10^2^) by ELISA against rOspA_B31_. In contrast to the controls, serum from all mice vaccinated with OspA collected before (D17) and after the boost (>D86) had anti-OspA IgG antibody OD_450_ > 3.5. To quantify anti-OspA IgG endpoint titers we used serum from Study 3 (15-month challenge) collected at day 17 (preboost), and at months 3, 6 and 12 post-prime (Fig. 4) diluted at 10^2^-10^6^ on ELISA. Mice from the s.c. rOspA_B31_+Alum, intranasal PIV5-A_BPBPk_, and intranasal PIV5-A_B31_ vaccine groups had peak serum IgG endpoint titers (EPT) at 3-month postprime, with geometric means of 5.5, 5.5 and 5.4 log_10_, respectively, and not statistically different between groups. These values were significantly higher than serum IgG EPTs from day 17 post-prime, showcasing the boosting effect by the second dose of the vaccine. These levels of anti-OspA_B31_ serum IgG antibodies decreased over the next 9-months, with mice from the s.c. rOspA_B31_+Alum group having the biggest decrease of 1.7 log_10_ EPT compared to 0.7 log_10_ EPT seen in mice from the intranasal PIV5-A_BPBPk_ and PIV5-A_B31_ vaccine groups. Mice from the PBS+Alum and PIV5 control groups had low levels of cross-reactive IgG antibodies throughout the study. As expected, analyses between intranasally vaccinated groups as well as between subcutaneously vaccinated groups show significant EPT differences between the controls (PIV5, Alum) and the groups of mice vaccinated with OspA (PIV5-A_BPBPk_, PIV5-A_B31,_ rOspA_B31_+Alum), *p* < 0.0001. Of note, between OspA vaccinated groups, EPT differences between intranasally delivered PIV5-A_BPBPk_ and PIV5-A_B31_ are not significant at 6- and 12-months post prime, but the differences in EPTs between rOspA_B31_+Alum delivered subcutaneously (which are lower) and the intranasal vaccines (PIV5-A_BPBPk_ and PIV5-A_B31_) are significant (*p* = 0.0116 and *p* = 0.0001 for 6-months, and *p* = 0.0008 and *p* = 0.0005 for 12-months, respectively). These data indicate that immunization with OspA-based intranasal PIV5 leads to longer-lasting production of anti-OspA antibody than a recombinant OspA protein-based vaccine given subcutaneously.

**Fig. 5:**
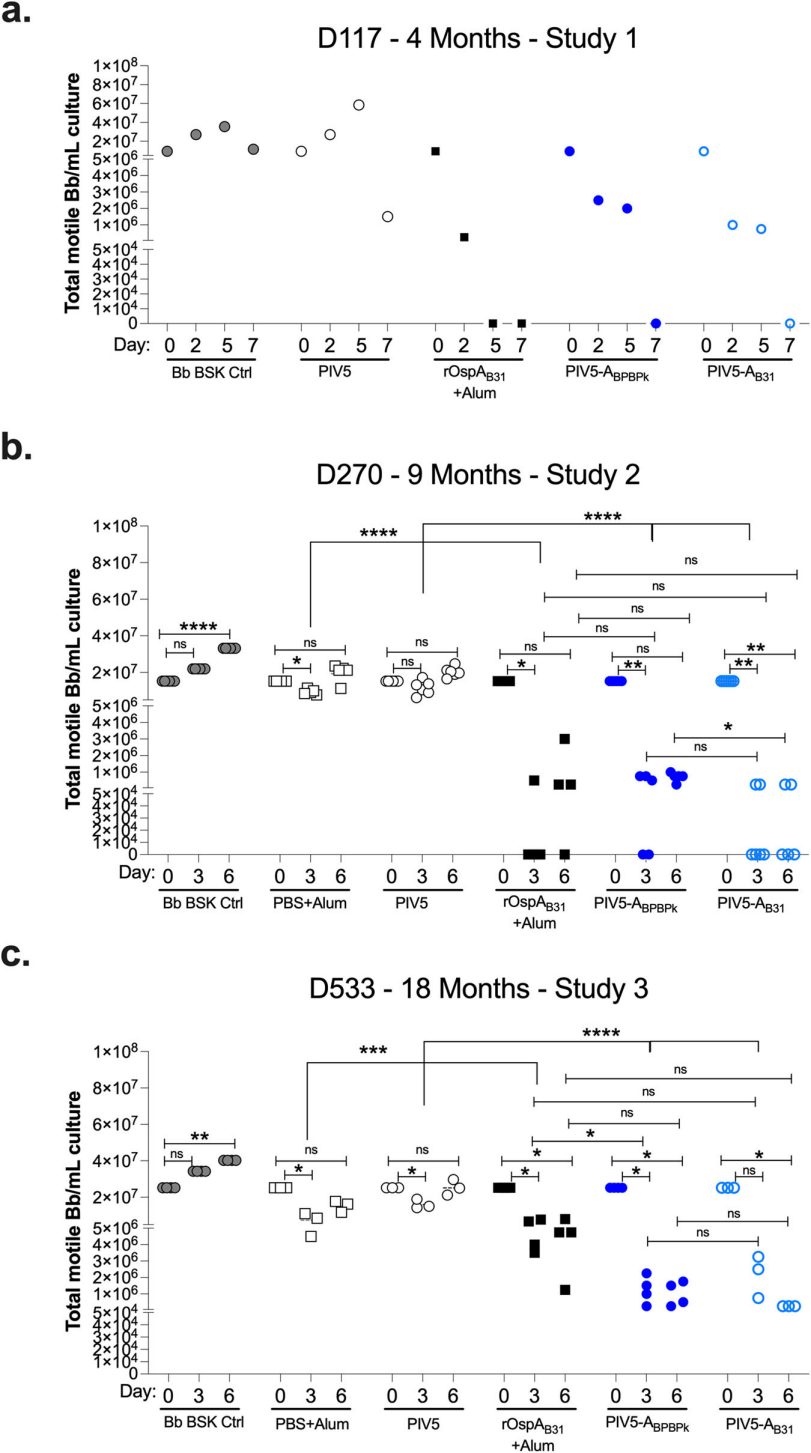
Intranasal vaccination with PIV5-A_BPBPk_ and PIV5-A_B31_ induces neutralizing antibodies up until 18-months post vaccination. Cultures of *B. burgdorferi* were treated with serum collected from mice before tick challenge at D117 (Study 1, **a**), and from unchallenged mice collected at D270 (Study 2, **b**) and D533 (Study 3, **c**). Neutralization was determined by counting motile spirochetes under a dark field microscope. Serum from Study 1 (4-months, D117) was pooled per group (*n* = 5 mice/group). For the subsequent Study 2 (9-month, D270) and Study 3 (18-month, D533), serum from *n* = 3–6 mice per group were individually tested. Scatter plots represent the average numbers of motile *B. burgdorferi* in 5 fields (Petroff-Hausser) counted under a dark field microscope. Statistical significance was calculated by Repeated Measures or Mixed Model 2-Way ANOVA (with Geisser-Greenhouse correction) for comparisons within routes of immunization, i.e. intranasal PIV5 v PIV5-A_BPBPk_ v PIV5-A_B31_ and subcutaneous Alum v OspA_B31_ +Alum. Differences between two OspA vaccinated groups were analyzed by multiple Mann-Whitney tests at each timepoint except day 0. The Kruskal-Wallis was used to test for differences between timepoints within each group (ns, not significant, *p < 0.05, **p < 0.005, ***p < 0.0005, ****p < 0.0001), Bb, *Borrelia burgdorferi*.

Similar to Study 3, mice from Study 2 (9-month challenge) were immunized with either two-doses of alum alone (s.c. PBS+Alum) or alum plus 20 µg of rOspA protein (s.c. rOspA_B31_+Alum) subcutaneously, or with two-doses of 10^6^ PFU of PIV5, PIV5-A_BPBPk_, or PIV5-A_B31_ intranasally (Fig. 4). Study 1 (4-month challenge) contained the same groups, except for the s.c. PBS+Alum which was an additional control deemed redundant at that stage of vaccine development. Neutralization assays (Fig. 5) were conducted using blood collected before tick challenge at D117 (Study 1), and from unchallenged mice at D270 (Study 2) and D533 (Study 3). Due to insufficient volumes of blood from Study 1, serum was pooled by group, while individual mouse samples were used for Studies 2 and 3. Total motile bacteria were counted in five fields in a Petroff-Hausser chamber under a dark field microscope on days 0, 2, 5, and 7 for Study 1, and days 0, 3, and 6 for Studies 2 and 3. In contrast to the controls, at 4-months after vaccination (Study 1, Fig. 5a), the numbers of motile *B. burgdorferi* in cultures incubated with serum from the rOspA_B31_+Alum, PIV5-A_BPBPk_, and PIV5-A_B31_ groups decreased by 1.6, 0.6, and 1.0 log_10_, respectively, at day 2 compared to day 0. These values further decreased on day 5 until they reached 0 for all vaccine groups at day 7. At 9-months after vaccination (Study 2, Fig. 5b), in control groups, *B. burgdorferi* cultures in BSK-H media grew a modest 0.2 log_10_, and numbers of *B. burgdorferi* in cultures treated with PBS+Alum and PIV5 serum did not increase from day 0 until day 6 post-neutralization. However, numbers of motile bacteria in cultures treated with serum from mice that received OspA (rOspA_B31_+Alum, PIV5-A_BPBPk_, and PIV5-A_B31_) were reduced at day 3 (~2 to 7 log_10_) and day 6 postneutralization (~1 to 7 log_10_) compared to day 0. Differences between each OspA vaccinated group and the respective control are significant (*p* < 0.0001). Differences between the three OspA vaccinated groups in terms of bacterial motility were only significant between PIV5-A_BPBPk_ and PIV5-A_B31_ at day 6 (*p* = 0.0238). At 18-months postvaccination (Study 3, Fig. 5c), the data continues to show a reduction in numbers of motile *B. burgdorferi* per milliliter of culture in samples from the rOspA_B31_+Alum, PIV5-A_BPBPk_, and PIV5-A_B31_ vaccine groups at days 3 and 6 post-neutralization compared to day 0, with the largest significant differences seen on day 6 with an average decrease of 0.8, 1.5 and 2.0 log_10_, respectively. As observed in Study 2 in the control groups, *B. burgdorferi* cultures in BSK-H media grew 0.2 log_10_, and numbers of *B. burgdorferi* in cultures treated with PBS+Alum and PIV5 serum did not increase from day 0 until day 6 post-neutralization. Differences between each OspA vaccinated group and the respective control are significant (*p* < 0.0001). Differences between the three OspA vaccinated groups in terms of bacterial motility were only significant between rOspA_B31_+Alum and PIV5-A_BPBPk_ with less numbers of motile bacteria counted in the latter, at day 3 post-neutralization (*p* = 0.0285). These results show that an intranasal PIV5-based vaccine carrying OspA sequences from different *B. burgdorferi* sensu lato genospecies can generate neutralizing IgG immune responses that lasted until 18-months post vaccination.

### PIV5-A_BPBPk_ and PIV5-A_B31_ provide long-term protection against tick challenge with multiple strains of *B. burgdorferi*, up to 15-months post prime-boost immunization

**Fig. 6:**
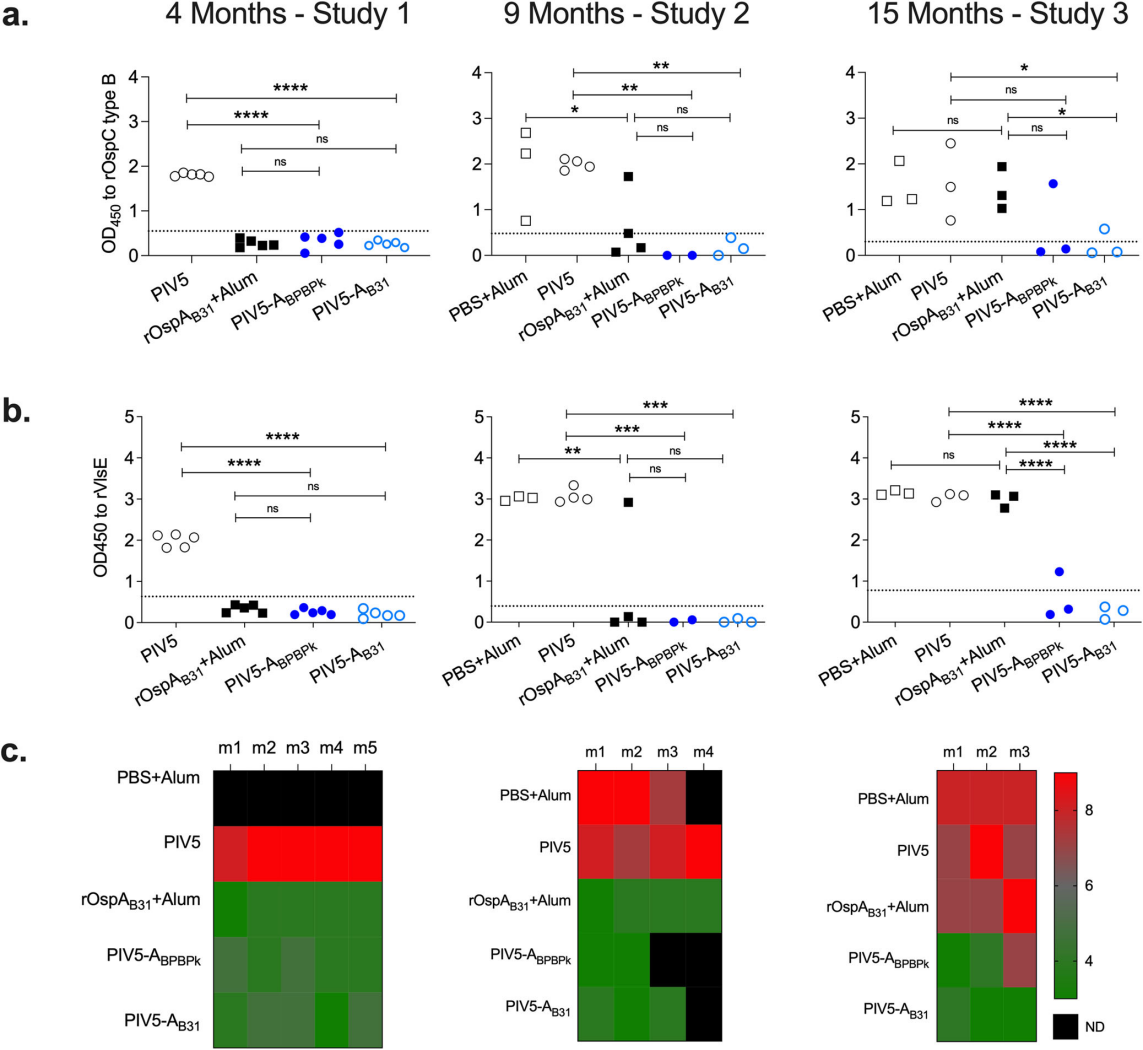
Serologic evidence of *B. burgdorferi* infection after tick challenge at 4-months, 9-months and 15-months post vaccination. IgG to *B. burgdorferi* was determined by assessment of serum antibody to recombinant OspC_B_ and VlsE by ELISA, as well as Western blot. Scatter dot plots (OD_450_) are shown for serum IgG to rOspC_B_ (**a**) and to rVlsE (**b**). Western blot data (**c**) is represented by heat maps of the enumerated bands: > 5 out of 10 bands (red, positive) and < 5 out of 10 bands (green, negative), m, mouse. Statistical significance was calculated by One-way ANOVA followed by multiple comparisons with uncorrected Fisher’s LSD (ns, not significant, *p < 0.05, **p < 0.005, ***p < 0.0005, ****p < 0.0001).

IgG to *B. burgdorferi* protein was quantified in serum from tick-challenged mice by ELISA (Figs. 6a, b) and Western blot (Fig. 6c). In Study 1, three weeks after the 4-month challenge, none of the mice vaccinated with OspA vaccines (rOspA_B31_+Alum, PIV5-A_BPBPk_ and PIV5-A_B31_) had anti-OspC_B_ or anti-VlsE antibody OD_450_ values above the cutoff, in contrast to the PIV5 control. Furthermore, serum from all mice that received OspA vaccines was negative on Western blot (2-4/10 bands and negative for OspC), in contrast to the PIV5 control (8-9/10 bands and positive for OspC). In Study 2, three weeks after the 9-month challenge, none of the mice vaccinated with PIV5-A_BPBPk_ and PIV5-A_B31_ had anti-OspC_B_ or anti-VlsE IgG antibody OD_450_ values above the cutoff, whereas 1 mouse from the rOspA_B31_+Alum group (1/4) had anti-OspC_B_ and anti-VlsE IgG well above the cutoff. As observed in Study 1, the OspC_B_ and VlsE serologic results were largely confirmed by Western blot, except one mouse vaccinated with OspA_B31_+Alum that although positive on ELISA was negative on Western blot. Last, in Study 3, three weeks after the 15-month challenge, all mice from the rOspA_B31_+Alum group (3/3), and 1/3 mice from PIV5-A_BPBPk_ and from the PIV5-A_B31_ groups had levels of anti-OspC_B_ IgG above the cutoff. Regarding anti-VlsE IgG, all mice from the rOspA_B31_+Alum group (3/3), and 1/3 mice from PIV5-A_BPBPk_ group had levels of IgG above the cutoff. In this study, the Western blot results largely supported the OspC_B_+VlsE IgG ELISA. Differences between controls and OspA vaccinated groups were significant in the three studies. Within the OspA vaccinated groups, significant differences in OspC_B_ and VlsE IgG serology were observed after the 15-month challenge: rOspA_B31_+Alum and PIV5-A_B31_ for OspC (*p* = 0.0427); rOspA_B31_+Alum and PIV5-A_BPBPk_ for VlsE (*p* < 0.0001); rOspA_B31_+Alum and PIV5-A_B31_ for VlsE (*p* < 0.0001).

**Fig. 7:**
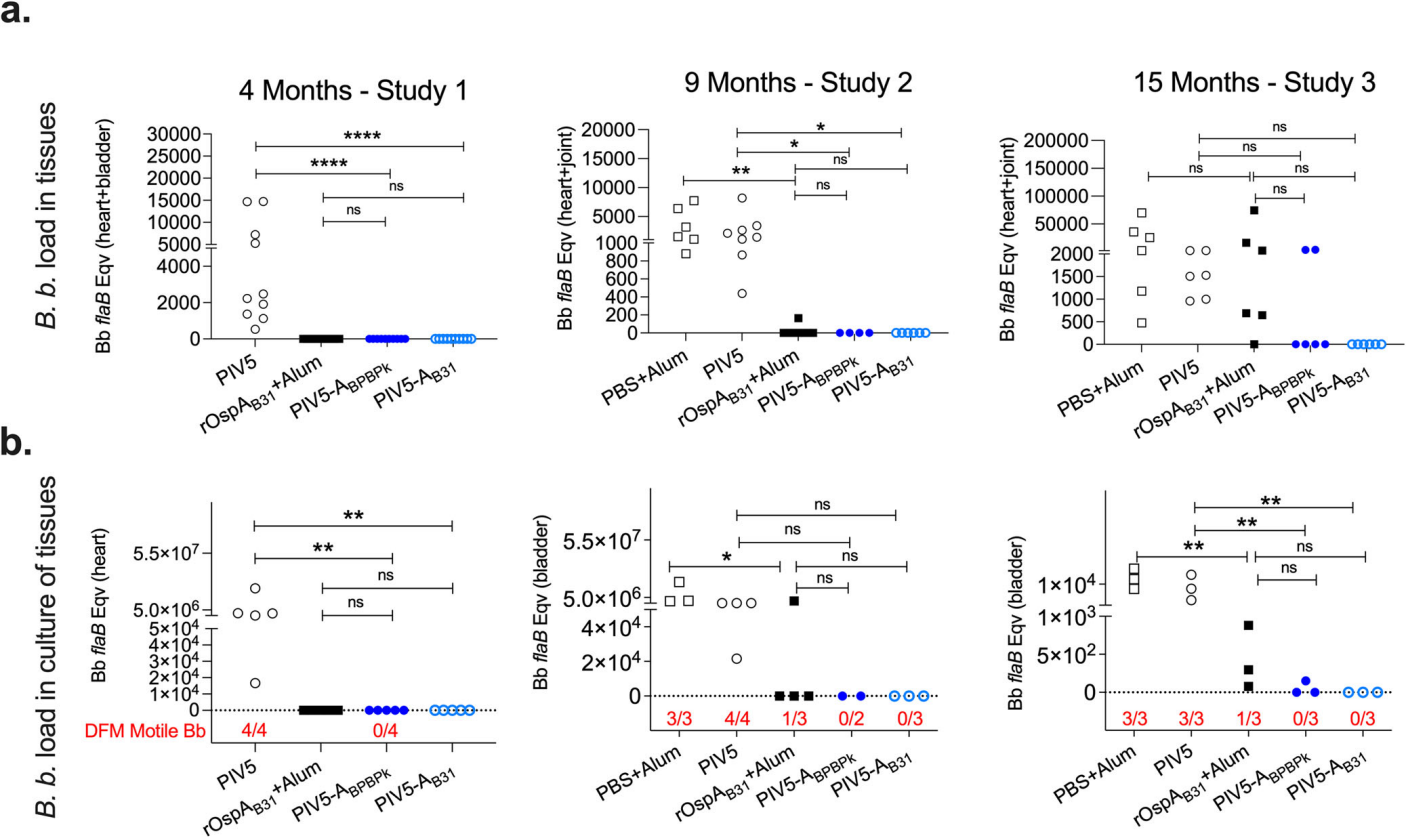
*B. burgdorferi* load and viability in mouse tissues after tick challenge at 4 months, 9 months and 15 months post vaccination. Tissues collected from vaccinated mice euthanized at 4-months (heart, bladder), and from mice euthanized at 9-months and 15-months (heart, joint) were processed for Bb DNA purification and *flaB* qPCR. Heart (4-months) and bladder (9- and 15-months) tissues were also cultured in BSK-H media for analysis of *B. burgdorferi* (Bb) growth and motility under a dark field microscope which was confirmed by *flaB* qPCR. Scatter dot plots are shown for Bb *flaB* load in tissues (**a**) and Bb *flaB* load in culture of tissues quantified (**b**) after assessment of *B. burgdorferi* motility from each culture by dark field microscopy - inset (**b**). Bb, *Borrelia burgdorferi*. Statistical analysis was calculated by multiple Mann-Whitney tests for 4 Months and by One-way ANOVA followed by multiple comparisons with Uncorrected Fisher’s LSD for 9 Months and 15 Months (ns, not significant, *p < 0.05, **p < 0.005, ****p < 0.0001).

Mice from the 3 studies were challenged with ticks infected with multiple strains of *B. burgdorferi* at 4-months (Study 1), 9-months (Study 2), or 15-months (Study 3) post-prime. To evaluate *B. burgdorferi* dissemination to target tissues, two tissues per mouse (heart, bladder, or joint) were tested for *B. burgdorferi flaB* load by qPCR (Fig. 7a). To assess *B. burgdorferi* viability, one tissue per mouse (heart or bladder) was cultured in BSK-H medium to evaluate growth and motility of *B. burgdorferi* under a dark field microscope (Fig. 7b, inset in red), which was confirmed by *flaB* PCR (Fig. 7b). In Study 1, the challenge done 4-months after vaccination with subcutaneous rOspA_B31_, and intranasal PIV5-A_BPBPk_ and PIV5-A_B31_ resulted in absence of *B. burgdorferi flaB* DNA in heart and bladder (Fig. 7a), as well as absence of viable *B. burgdorferi* in cultures from heart which was confirmed by *flaB* qPCR (Fig. 7b), in contrast to the controls that received intranasal PIV5. Differences between controls and OspA vaccinated groups are significant. In Study 2, the challenge done 9-months after vaccination with intranasal PIV5-A_BPBPk_ and PIV5-A_B31_ resulted in absence of *B. burgdorferi flaB* DNA in heart and joint (Fig. 7a), as well as absence of viable *B. burgdorferi* in culture from bladder which was confirmed by *flaB* qPCR (Fig. 7b). In contrast, 1 mouse that received subcutaneous rOspA_B31_+Alum had a positive joint *flaB* PCR which was confirmed by a positive culture from bladder. All controls that received subcutaneous PBS+alum or intranasal PIV5 had positive *flaB* PCR from heart and joint tissues, as well as PCR confirmed bladder cultures. Differences between controls and OspA vaccinated groups were significant for *B. burgdorferi flaB* load in tissues, and for amplification of *B. burgdorferi flaB* from culture of bladder in mice vaccinated with rOspA_B31_+Alum and its respective control. In Study 3, the challenge done 15-months after vaccination with intranasal PIV5-A_B31_ resulted in absence of *B. burgdorferi flaB* DNA in heart and joint (Fig. 7a), as well as absence of viable *B. burgdorferi* in cultures from bladder which was confirmed by *flaB* qPCR (Fig. 7B). Furthermore, one PIV5-A_BPBPk_ vaccinated mouse had ~ 3500 copies of *flaB* in joint as well as in heart tissue, which was not confirmed by growth and motility analysis from the bladder culture or *flaB* PCR from the same culture. In contrast, 5 tissues (3 joint, 2 heart) from the 3 mice vaccinated with rOspA_B31_+Alum had ~600-74,000 *flaB* DNA copies, PCR from the cultures of the 3 bladders had 77-800 *flaB* DNA copies, but only 1 bladder of the 3 mice produced a culture with motile *B. burgdorferi*. Regarding the controls, the 3 mice that received subcutaneous PBS+Alum and the 3 mice that received intranasal PIV5 had all joint and heart tissues positive for *flaB* PCR and all the bladders from each mouse produced cultures with motile *B. burgdorferi*, that were confirmed by PCR. In Study 3, differences between controls and OspA vaccinated groups were significant for *B. burgdorferi flaB* in culture of tissues.

The vaccine efficacy data is summarized in Table 1 and includes anti-*B. burgdorferi* Western blot serology, *B. burgdorferi flaB* load in tissues, *flaB* load in culture of tissues and *B. burgdorferi* motility under dark field microscopy after tick challenge.

**Table 1:** Vaccine efficacy data summary.

Study	Groups	No. of mice challenged (8-10 ticks per mouse)	B.b. W-blot serology	*flaB* load in tissues (2 per mouse)	*flaB* load in culture (1 tissue/mouse)	B.b. motility by DFM
			No. P/T (%)	No. P/T (%)	No. P/T (%)	No. P/T (%)
Study 1 -4 Month Chlg (*n* = 5 mice/gr)	PIV5 IN	5	5/5 (100)	10/10 (100)	5/5 (100)	4/4 (100)
	rOspA_B31_+Alum SC	5	0/5 (0)	0/10 (0)	0/5 (0)	nd
	PIV5-A_BPBPk_ IN	5	0/5 (0)	0/10 (0)	0/5 (0)	0/4 (0)
	PIV5-A_B31_ IN	5	0/5 (0)	0/10 (0)	0/5 (0)	nd
Study 2 -9 Month Chlg (*n* = 10 mice/gr)	PBS+Alum SC	3	3/3 (100)	6/6 (100)	3/3 (100)	3/3 (100)
	PIV5 IN	4	4/4 (100)	8/8 (100)	4/4 (100)	4/4 (100)
	rOspA_B31_+Alum SC	4	0/4 (0)	1/8 (12.5)	1/4 (25)	1/3 (33)
	PIV5-A_BPBPk_ IN	3	0/2 (0)	0/4 (0)	0/2 (0)	0/2 (0)
	PIV5-A_B31_ IN	4	0/3 (0)	0/6 (0)	0/3 (0)	0/3 (0)
Study 3 -15 Month Chlg (*n* = 10 mice/gr)	PBS+Alum SC	3	3/3 (100)	6/6 (100)	3/3 (100)	3/3 (100)
	PIV5 IN	3	3/3 (100)	6/6 (100)	3/3 (100)	3/3 (100)
	rOspA_B31_+Alum SC	3	3/3 (100)	5/6 (83.3)	3/3 (100)	1/3 (33)
	PIV5-A_BPBPk_ IN	3	1/3 (33)	2/6 (33)	1/3 (33)	0/3 (0)
	PIV5-A_B31_ IN	3	0/3 (0)	0/6 (0)	0/3 (0)	0/3 (0)

*B.b*. *Borrelia burgdorferi,* *IN* intranasal (10^6^ PFU), *SC* subcutaneous (20 µg), *M* month, *gr* group, *Chlg* challenge, *PFU* plaque forming units, *W-blot* western blot, *DFM* dark field microscopy, *No.* *P/T* Number of Positive/Total, *nd* not determined. Differences in number of mice and data acquired over time was due to loss of aged mice.

**Fig. 8:**
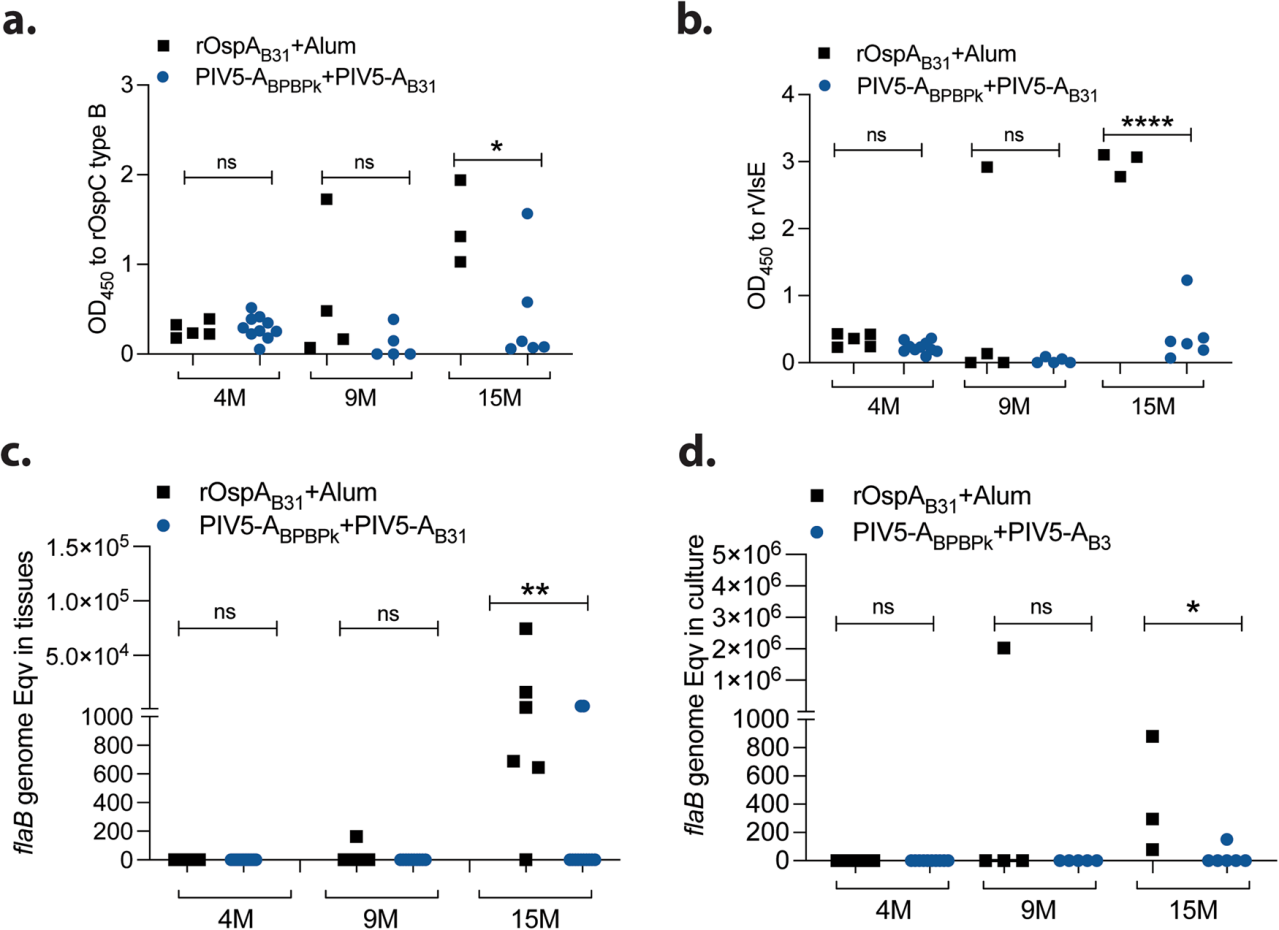
Vaccine efficacy at 4-months, 9-months and 15-months, by route of administration. Anti-*B. burgdorferi* OspC (**a**) and VlsE (**b**) antibody in serum, as well as *B. burgdorferi flaB* DNA amplified from tissues (**c**) and from culture of tissues (**d**) was compared between subcutaneous (s.c.) and intranasal (i.n.) routes of immunization with OspA. Statistics by Welch’s t test (**a**, **b**) and Mann Whitney (**c, d**) between s.c. and i.n. groups, *p < 0.05, ** *p* < 0.005, ****p < 0.0001. M, months.

We lost mice throughout our study in all groups subjected to the 9-month and 15-months protocols due to attrition. This impacted vaccine efficacy analysis of each PIV5-OspA vaccine group. To evaluate if the vehicle and route of immunization affected vaccine efficacy, we analyzed differences between groups of mice vaccinated subcutaneously with OspA_B31_+Alum and mice vaccinated intranasally with PIV5 carrying OspA (A_BPBPk_+A_B31_) (Fig. 8). We found that differences in anti-OspC_B_ (Fig. 8a) and anti-VlsE (Fig. 8b) IgG between subcutaneous rOspA_B31_+Alum and the intranasal immunizations (PIV5-A_BPBPk_ + PIV5-A_B31_) are significant after the 15-month challenge (*p* = 0.0374 and *p* < 0.0001, respectively). The same was observed for differences in *B. burgdorferi flaB* DNA in tissues (Fig. 8c) and in culture from tissue (Fig. 8d) between subcutaneous rOspA_B31_+Alum and the PIV5-A_BPBPk_ + PIV5-A_B31_ intranasal immunizations (*p* = 0.0093 and *p* = 0.0238, respectively). Furthermore, a combined analysis of motile *B. burgdorferi* in culture from tissues in the 9 M and 15 M challenges (Fig. 7b inset, and Table 1) shows that 2/6 (33%) mice vaccinated subcutaneously with OspA_B31_+Alum produced positive culture results, which stands in stark contrast to 0/5 (0%) and 0/6 (0%) mice vaccinated intranasally with PIV5-A_BPBPk_ and PIV5-A_B31_, respectively.

Together, these data demonstrate that an intranasal PIV5-vectored OspA-based Lyme disease vaccine can provide substantial longer-lasting protection than a recombinant protein-based OspA vaccine given subcutaneously to mice.

## Discussion

Currently, the most effective way to prevent Lyme disease is to avoid *I. scapularis* tick infested areas. This is unfeasible for those who work outside and for those who enjoy spending quality time outdoors in the Spring and Summer in endemic areas. Thus, there is a pressing need for development and commercialization of effective and acceptable vaccines to control Lyme disease. We developed a parainfluenza virus 5 (PIV5) viral-vectored vaccine for intranasal delivery of OspA that provides mice with long-lasting protection against tick-transmitted *B. burgdorferi* using a prime-boost scheme of immunization.

Outer surface protein A from *B. burgdorferi* sensu stricto (OspA) is the only immunogen proven to provide high (LYMErix^TM^)^2^ or very high (ImuLyme^TM^)^3^ protection against tick-transmitted *B. burgdorferi* in human subjects after 3 intramuscular injections. One of the two vaccines (LYMErix^TM^) was approved by the FDA in 1998. Although analysis of adverse effects performed in both clinical trials showed no significant increase in the frequency of arthritis events between vaccine and control groups^2,3^, some individuals who received LYMErix, reported developing arthritis after the trial period ended^20^. Furthermore, within weeks of the clinical trial reports, another study suggested that a cross-reactive autoimmune event between an epitope in *B. burgdorferi* OspA and a human integrin (hLFA-1) might drive the inflammatory response in the joints of some treatment-resistant Lyme arthritis patients^21^. Even though LYMErix was never linked to causing arthritis, demand for this vaccine decreased substantially and the product was taken off the market in 2002^22^. Keeping all these factors in mind, we designed a chimeric sequence of OspA by replacing the hLFA-1 partially homologous epitope within *B. burgdorferi* OspA with the analogous sequence from *B. afzelii* (a nonarthritogenic *Borrelia* species) to generate the new OspA_BPBPk_ construct. This sequence was cloned into a Lactobacillus expression vector and was shown to prevent tick-transmitted *B. burgdorferi* infection in mice, thus providing an effective oral vaccine candidate for Lyme disease^18^.

Parainfluenza virus 5 (PIV5) is a nonsegmented, negative-strand, RNA virus and a member of the *Rubulavirus* genus of the family *Paramyxoviridae*. PIV5 is a promising safe viral vaccine vector. Live PIV5 has been part of the kennel cough vaccine for dogs for 50 years, yet no disease has ever been reported due to human exposure to vaccinated dogs^23^. We have not performed studies looking at transmission of PIV5 from vaccinated to naïve individuals. Nevertheless, we found that ~30% of humans have anti-PIV5 antibodies, suggesting that humans can get infected with PIV5 from PIV5-vaccinated dogs^24^. In that study we also show that the anti-PIV5 pre-existing antibody did not affect the immunogenicity of the PIV5-vectored vaccine^24^.

PIV5 does not have a DNA phase in its life cycle. Thus, its use avoids the unintended consequences of genetic modifications of host cell DNA through recombination or insertion. Various recombinant PIV5 viruses expressing GFP or immunogens have been generated and shown to be genetically stable^25,26^. Furthermore, a study performed with a PIV5-based influenza vaccine in nude mice showed no sign of illness or weight loss, as well as no enhanced pathology, indicating that PIV5 is safe and non-pathogenic in an immune deficient mouse model^27^. In addition, PIV5 can grow in Vero cells at titers greater than 10^8^ PFU/mL, making vaccine production cost effective. PIV5-based influenza (IAV)^19,27–31^, respiratory syncytial virus (RSV)^12,26,32^, MERS-CoV^33^ and, most recently SARS-CoV-2^34^, vaccines are efficacious in various preclinical animal models. RSV and COVID-19 vaccines have recently completed phase 1 clinical trials.

In this study, we demonstrate that both PIV5-based vaccines, PIV5-A_BPBPk_ and PIV5-A_B31_, administered in a homologous prime-boost vaccination regimen intranasally, can induce robust and long-lasting humoral IgG immune responses in mice reaching over 5 log10 EPT for serum IgG antibodies at 3-months post-vaccination, with these values moderately decreasing up to 12-months post-immunization. Although the data shows a significant increase in anti-OspA_B31_ EPTs in serum from mice that received the intranasal vaccines (PIV5-A_BPBPk_ and PIV5-A_B31_) compared to the subcutaneous control (rOspA_B31_+Alum) at 6-months and at 12-months postprime-boost (Fig. 4) the ultimate functionality of the vaccine could be related to other factors such as affinity maturation and glycosylation. However, we have not performed studies to determine if glycosylation and affinity maturation play a role in the immunogenicity of PIV5-based vaccines.

The longevity of the immune response to the PIV5 delivered OspAs can be noticed in Fig. 5, where differences in neutralization of *B. burgdorferi* with mouse serum favors PIV5-A_BPBPk_ at 18-months post-immunization. One of the advantages of using live replicating viral vectored vaccines is that IgG responses are more durable^35^ than responses induced by mRNA vaccines which usually wane within 6 months to 1 year^36^. For OspA recombinant proteins, others have shown that OspA-specific IgG antibody also starts to decline 3-6 months after the 2^nd^ or 3^rd^ dose within the first year of vaccination^37^. Other schedules of administration (3 shots within 2 months) produced anti-OspA antibody responses that could only be protective for one tick-season (about 4 months)^38^. Furthermore, Comstedt et al., reported that immunization with 3 doses of VLA15 in mice resulted in a robust serum IgG antibody response, but the levels of antibodies decreased more than 10-fold, five months after immunization^9^. A booster dose was administered 5 months after immunization to increase these levels. To accommodate these issues, the VLA15 vaccine undergoing phase 3 clinical trials requires a 3-shot intramuscular vaccination schedule on Day 1 (Month 0), Day 57 (Month 2) and Day 180 (Month 6). Here, we show that prime-boost immunization with a mucosal delivered live viral-vectored vehicle leverages a non-invasive administration route (intranasal) and generates a rapid, durable and neutralizing IgG response to the immunogen that lasts up to 18-months.

Longevity of protection was associated with PIV5 delivered OspA vaccinations in challenges performed at 9- and 15-months post-prime (Figs. 6, 7, 8, Table 1). Our comparative analysis of serologic anti-OspC and anti-VlsE IgG antibodies, as well as presence of *B. burgdorferi flaB* DNA in tissues and in culture from tissues shows significant differences between mice subcutaneously vaccinated with rOspA_B31_ and mice vaccinated with both PIV5 delivered OspA_BPBPk_ and OspA_B31_ in groups challenged at 15-months after vaccination. Furthermore, we determined that 33% of mice vaccinated subcutaneously with OspA_B31_+Alum had motile *B. burgdorferi* in culture from tissues, whereas none of the mice vaccinated intranasally with PIV5-A_BPBPk_ and PIV5-A_B31_ produced cultures with motile bacteria after the 9 M and 15 M challenges. Taken together, our data indicates that longevity of vaccine efficacy is consistently higher in mice that received intranasal PIV5 delivered OspA vaccines compared to mice that received parenteral OspA vaccine.

Of note, in challenges done at 9-months post vaccination, the mouse vaccinated with rOspA_B31_+Alum that developed IgG antibodies to OspC and VlsE had a negative Western blot (Fig. 6). However, it also produced a positive *flaB* result in joint, a positive *flaB* bladder culture and motile *B. burgdorferi* in the same culture under dark field microscopy (Fig. 7). In challenges done at 15-months post vaccination, serologic IgG to OspC and VlsE in the mouse vaccinated with PIV5-A_BPBPk_ was further supported by a positive Western blot result (Fig. 6). However, although this mouse produced positive tissues for *flaB* DNA, *B. burgdorferi* did not grow in culture (Fig. 7). Similar data was observed for mice vaccinated with OspA_B31_+Alum challenged at 15-months post vaccination, in that the 3 mice had antibodies to OspC and VlsE which were supported by Western blot. However, although the 3 mice produced positive *flaB* results in tissues, only 1 produced a culture with motile *B. burgdorferi*. These data suggest that a weaker immune counter activity at 15-months possibly due to lower or impaired function of myeloid and lymphoid cells may allow persistence of viable *B. burgdorferi* and *B. burgdorferi* debris in some target tissues. The data also lends credence to the difficulties with development of diagnostic assays for Lyme disease by currently available serologic or molecular methods.

We developed a PIV5-vectored OspA-based intranasal vaccine that prevents tick-transmitted *B. burgdorferi* infection in mice challenged at 4-, 9- and 15- months post prime-boost immunization. Our work advances the field of development of vaccines for Lyme disease in three ways: i) it reduces the number of immunizations, ii) it simplifies administration from the classic intramuscular injection to an intranasal mist with possibility for self-administration, iii) and most importantly, it extends protection until 15-months post-immunization.

## Methods

### Construction of PIV5-vectored vaccines expressing OspA_B31_ and OspA_BPBPk_

#### Cells

BHK21 cells were maintained in Dulbecco’s modified Eagle medium (DMEM) containing 10% tryptose phosphate broth (TPB), 5% fetal bovine serum (FBS), 100 IU/mL penicillin, and 100 µg/mL streptomycin (1% P/S; Mediatech Inc., Manassas, VA). Vero E6 and MDBK cells were maintained in Dulbecco’s modified Eagle media (DMEM) supplemented with 5% fetal bovine serum (FBS) plus 100 IU/mL penicillin and 100ug/mL streptomycin (1% P/S; Mediatech Inc, Manassas, VA, USA). All cells were incubated at 37°C, 5% CO_2_.

#### Viruses

The PIV5-A_B31_ and PIV5-A_BPBPk_ plasmids, encoding the full-length genome of PIV5 and a *Borrelia burgdorferi* OspA gene (strain B31) and well as a chimeric OspA gene comprised of sequences from *B. burgdorferi* B31, and *B. afzelii* PGau and Pko (B31_1–164_PGau_165–189_B31_190–218_Pko_219–273_ - BPBPk)^18^ inserted between the PIV5 small hydrophobic (SH) and hemagglutinin-neuraminidase (HN) genes was constructed as previously described^19^. Briefly, the PIV5-A_B31_ and PIV5-A_BPBPk_ plasmids and four helper plasmids—pPIV5-NP, pPIV5-P, pPIV5-L, and pT7-polymerase, encoding the NP, P, and L proteins and T7 RNA polymerase, respectively—were co-transfected into BHK21 cells at 90% confluence n 6-cm plates using JetPrime (Polyplus). Recovery of the virus is indicated by syncytia formation. The virus was then plaque-purified as a single plaque from BHK21 cells. The full-length genomes of the plaque-purified single clone of PIV5-A_B31_ and PIV5-A_BPBPk_ viruses were sequenced as described^19^. Viruses were grown in MDBK cells for 5 to 7 days using DMEM containing 2% fetal bovine serum (FBS). Media were collected and pelleted at 500 x*g* to remove cell debris by using a Sorvall tabletop centrifuge for 10 min. Virus supernatant was supplemented with 10% sucrose-phosphate-glutamate buffer, snap-frozen in liquid nitrogen, and stored at −80°C immediately after collection.

#### Western blot

Immunoblotting was performed on Vero cells in 6-well plates that were infected with PIV5, PIV5-A_B31_, or PIV5-A_BPBPk_ at an MOI of 1. At 48 hours post-infection (hpi), Laemmli sample buffer (Bio-Rad, catalog no. 1610737) with 5% β-mercaptoethanol was used to lyse cells. The lysates were separated on an SDS–polyacrylamide gel electrophoresis (SDS-PAGE) gel and immunoblotted with a mouse anti–OspA monoclonal antibody (184.1, 1:100) and an anti–PIV5-NP monoclonal antibody (NP214mAb, 1:100, a kind gift from Dr. Randall to Dr. He).

All blots were processed in parallel and derive from the same experiments.

### Immunization, immunogenicity, and vaccine efficacy analyses

All experiments were performed in accordance with the National Institutes of Health Guide for the Care and Use of Laboratory Animals under protocols approved by the Institutional Animal Care and Use Committee (IACUC) of the University of Georgia (A2023 01-021-Y1-A0) and the University of Tennessee Health Science Center (19-0103). A graphic representation on the experimental design is shown in Fig. 3.

#### Immunization

Briefly, groups of 5 to 8-week-old female C3H-HeN mice (Envigo) were anesthetized and intranasally inoculated with 50 µL of 10^6^ PFU of PIV5 vector, PIV5-A_B31,_ or PIV5-A_BPBPk,_ or with 100 µL of 20 µg of rOspA_B31_+Alum subcutaneously (Fig. 3). Twenty-one days after prime immunization, the mice were boosted with the same preparations. Blood was collected before tick challenge for determination of anti-OspA antibody on the following days: d17, d86, d100 and d117 for Study 1 (4-month challenge, D117 serum was used for neutralization assays); d17, d88, d103, d118 and monthly thereafter until d270 for Study 2 (9-month challenge, D270 serum was used for neutralization assays). For Study 3 (15-month challenge and 18-month neutralization), groups of mice were bled on d17, d86, d100 and d117 and monthly thereafter until D455 for 15-month challenge; a subset of these mice were not challenged and were kept until 18-months post prime-boost for an additional collection of blood at D533 for analysis of anti-*B. burgdorferi* neutralization activity.

#### Tick challenge

Three colonies of infected *I. scapularis* were maintained in the laboratory. MS’08/NY derived from cultures of tissue from *Peromyscus leucopus* infected with field caught NY ticks between 2005 and 2008 and frozen at -80C; MS’21/MA derived from frozen cultures obtained from C3H-HeN mice inflected with ticks collected in MA parks in 2021; MS’21/NY derived from frozen cultures from C3H-HeN mice inflected with ticks collected in NY parks in 2021. Cultures were used to infect C3H-HeN mice with 50,000 *B. burgdorferi* in 100-200 *µ*l; uninfected larval ticks were allowed to engorge in the infected mice and were allowed to molt to the nymphal stage before they were used for challenge. Challenge of experimental animals with *B. burgdorferi* was performed as described^39^. Ticks from the MS’08/NY colony were used for the 4-month challenge experiment; equal numbers of ticks from each 2021 colony (MS’21/MA and MS/21/NY) were used for the 9-month and 15-month challenges. The *B. burgdorferi* infection prevalence of each tick colony was determined to be >80% by *flaB* qPCR. Briefly, 8-10 flat nymphal *Ixodes scapularis* ticks were placed between the ears of mice that were caged separately and held in FIC-2 isolators for a week. Engorged ticks that naturally fell off after taking a bloodmeal were collected from the bottom of the cage, counted, labeled, and stored at -20°C. Three weeks after the last day of challenge, mice were euthanized and blood, heart, joint and bladder were collected. Tissues were placed in BSK-H culture or RNAlater (Invitrogen, MA) for determination of *B. burgdorferi* growth and motility (counting live spirochetes under a dark field microscope) and *B. burgdorferi* presence in tissues and in culture of tissues was done by *flaB* qPCR. Blood was used for analysis of anti-*B. burgdorferi* antibodies by ELISA.

#### Experimental endpoints

1) euthanasia of vaccinated mice for collection of blood for neutralization assays on D270 for Study 2 and on D533 for Study 3; 2) euthanasia of mice previously vaccinated and challenged, 3 weeks after the last day of challenge, for collection of blood and tissues for analyses of vaccine efficacy on D146 for Study 1, on D333 for Study 2 and on D494 for Study 3. Methods of anesthesia (inhalation of 3% isoflurane) and euthanasia (inhalation of 3-4% isoflurane, followed by exsanguination and thoracotomy for collection of tissues) were consistent with the recommendations of the American Veterinary Medical Association (AVMA) Guidelines.

#### Enzyme-linked immunosorbent assay (ELISA)

Anti-OspA, anti-OspC type B (OspC_B_) and anti-VlsE antibody was determined by ELISA^40,41^. Briefly, 5-10 µg/ml of purified recombinant OspA, OspC_B_ or VlsE were coated on flat-bottom ELISA plates (Thermo Fisher Nunc MaxiSorp) and incubated at 4°C overnight. The following day, the plates were washed, blocked, and incubated with primary antibodies, i.e., serum (1:100 or 1:1000), followed by the secondary antibody HRP-conjugated goat anti-mouse IgG (Jackson ImmunoResearch, Inc, cat. no.115-035-146) diluted at 1:10000. Endpoint titers of anti-OspA antibodies were calculated using serum (1:10^2^ to 1:10^6^) from study 3 (15-month challenge) collected at pre-boost, 3-months, 6-months and 12-months post-prime vaccination. Blood used for OspC_B_ and VlsE ELISA was collected at euthanasia on D146 (4-month challenge), D333 (9-month challenge) and D494 (15-month challenge). The ELISA cutoff was established at 5 standard deviations above the mean of 4 samples confirmed negative by Western blot.

#### Western blot

the *B. burgdorferi* IgG Virablot kit (Viramed Biotech AG, cat. no. V-BBSGUS) was used according to the manufacturer recommendations. A positive result was determined by observation of >5 out of 10 bands; a negative result was determined by observation of <5 out of 10 bands.

#### Neutralization antibody (nAb) assay

Briefly, blood was collected from groups of mice the day before challenge (d117) for the 4-month challenge experiment, and on days 270 and 533 from groups of mice that were not subjected to challenge for the 9-months and 18-months experiments, respectively. Neutralization of motility of multi-strain cultures of *B. burgdorferi* by fresh serum was performed as described^40^. Briefly, 8 µl of the bacterial culture was mixed with 4 µl of heat-inactivated mouse serum obtained from vaccinated and control mice and with 4 µl guinea pig complement (MP Biomedicals™) in a 0.2 ml sterile PCR microtube (VWR, LLC Radnor, PA). The positive control group consisted of 8 µl BSK media (Sigma-Aldrich, Saint Louis, MO) with 8 µl *B. burgdorferi* culture. Samples were incubated at 34°C for 6 to 7 days. The cultures were counted in five fields on days 0, 2, 5, 7 (4-month) or 0, 3, and 6 (9- and 18-month) for motile *B. burgdorferi* using a Petroff-Hausser chamber under a dark-filed microscope (Zeiss USA, Hawthorne, NY) and averaged to get the total number of motile bacteria.

#### Amplification of OspC by conventional PCR

Five flat nymphal *Ixodes scapularis* ticks were crushed and DNA was extracted using the DNAeasy tissue kit as per manufacturer’ recommendation (Qiagen, Valencia CA). OspC specific amplicons were prepared using primers (F)- AATAAAAAGGAGGCACAAATTAATG and (R)-GTAACTGGAAAAATAAAGTCAATAT by conventional PCR. Each 20 *µ*l PCR reaction mixture contained 200 *µ*M deoxynucleoside triphosphate (dNTP) (Thermo Scientific), 1 U Taq DNA polymerase (Thermo Scientific), 2 *µ*l of 10X Taq Buffer (Thermo Scientific), 2.5 mM MgCl_2_ and 0.4 *µ*M of each OspC primer, and 1 *µ*l of purified genomic DNA (gDNA). The reaction mixture was heated at 95°C for 4 min, amplified for 36 cycles at 95°C for 30 s, 58°C for 30 s, and 72°C for 60 s, and finally incubated at 72°C for 5 min. The PCR products (2-5 ul) were electrophoresed on a 2% agarose gel, pre-stained with 1:10,000 DNA SafeStain (Lambda Biotech-C138) and imaged under a UV gel doc system. AM-Pure beads were used to clean unused primers, dNTPs, and other reagents. The amplicon quantity was measured on a Qubit 4 fluorometer (Thermo Fisher Scientific, Waltham, MA, USA) using the Qubit dsDNA HS assay kit to ensure at least 10 ng/*µ*l for library preparation as per manufacturer’ recommendation. Amplicon quality was measured using a nanodrop instrument to confirm gDNA purity with A260/230 and A260/280 absorbance ratio of at least 1.8 for all samples. Library preparation was done using NEBNext® Fast DNA Fragmentation & Library Prep Set for Ion Torrent™. Samples were amplified and barcoded with barcode adapters from the Ion Xpress™ Barcode Adapters 1-16 Kit. Sequencing was done with the Proton Ion Torrent Sequencing instrument (ThermoFisher) at the UTHSC Molecular Resource Center.

#### OspC sequencing data analysis

Fastq files were retrieved from the Ion Torrent Sequencing instrument. The individual runs were combined to create the merged fastq files. FastQC was used to check the quality of the reads. Reads were trimmed if needed using FASTX-Trimmer. Data was aligned against the fasta file containing OspC variants provided. SAM files were mined for total read count tables. SAM files were then filtered to obtain only uniquely aligned fragments. Filtered SAM files were mined for unique read count tables. Graphs were created using R^42^ and Graphpad Prism.

#### Quantitative PCR to enumerate *B. burgdorferi*

Quantitative PCR (qPCR) was used to enumerate *B. burgdorferi* in BSK cultures from glycerol stock (used to infect mice), cultures from tissues from experimental mice and *B. burgdorferi* load in tissues and ticks used for challenge. Ticks and tissues (bladder, heart, joint) were processed for DNA extraction using the DNAeasy tissue kit as per the manufacturer’s recommendation (Qiagen, Valencia CA). The eluted DNA was stored at -20°C. qPCR was done on QuantStudio 3 (Applied Biosystems) using *B. burgdorferi flaB* primers, a known conserved gene of *B. burgdorferi*^43^. The following are the sequences: forward GCAGCTAATGTTGCAAATCTTTTC, reverse GCAGGTGCTGGCTGTTGA, and probe [6 ~ FAM]-AAACTGCTCAGGCTGCACCGG-[Tamra~Q]. For the standard curve, DNA from a *B. burgdorferi* culture from a stock of 10^6^ (determined by counting all visible *B. burgdorferi* under a dark field microscope) was purified and serially diluted from 10^5^ to 1. The PCR reaction was performed using the fast advance master mix (Applied Biosystems™ Taqman™) in a final 20 µl volume which contained 25 µM of each primer, 250 nM of the specific probe, and 2 µl of DNA.

### Statistical analysis

Data are represented in scatter dot plots. Statistical analysis was done by Repeated Measures or Mixed Model 2-Way ANOVA (with Geisser-Greenhouse correction) for comparisons within routes of immunization (Fig. 4, Fig. 5). Differences between two groups were analyzed by nonparametric multiple Mann-Whitney tests (Fig. 4, Fig. 5, Fig. 7, Figs. 8C, 8D) or unpaired Welch’s t test (Figs. 8A, 8B). The Kruskal-Wallis was used to test for differences between timepoints within each group (Fig. 5). One-way ANOVA followed by multiple comparisons with Uncorrected Fisher’s LSD was used to compare pairs of groups (Fig. 6, Fig. 7). GraphPad Prism was used for statistical analysis and plotting the graphs.

### Reporting summary

Further information on research design is available in the Nature Research Reporting Summary linked to this article.

### Supplementary information

Supplemental material

Reporting Summary

## Data Availability

All data generated or analyzed during this study are included in this manuscript. All relevant data are available from the authors.
