# Supplementary material for: Intranasal vaccine for Lyme disease provides protection against tick transmitted Borrelia burgdorferi beyond one year
Source: NPJ Vaccines. 2024 Feb 15;9:33. doi: 10.1038/s41541-023-00802-y (PMC10869809; doi:10.1038/s41541-023-00802-y)
Supplement: Supplementary file 1 — Supplemental material [file 41541_2023_802_MOESM1_ESM.pdf]

SUPPLEMENTARY Information - Uncropped Western Blot used in Figure 2

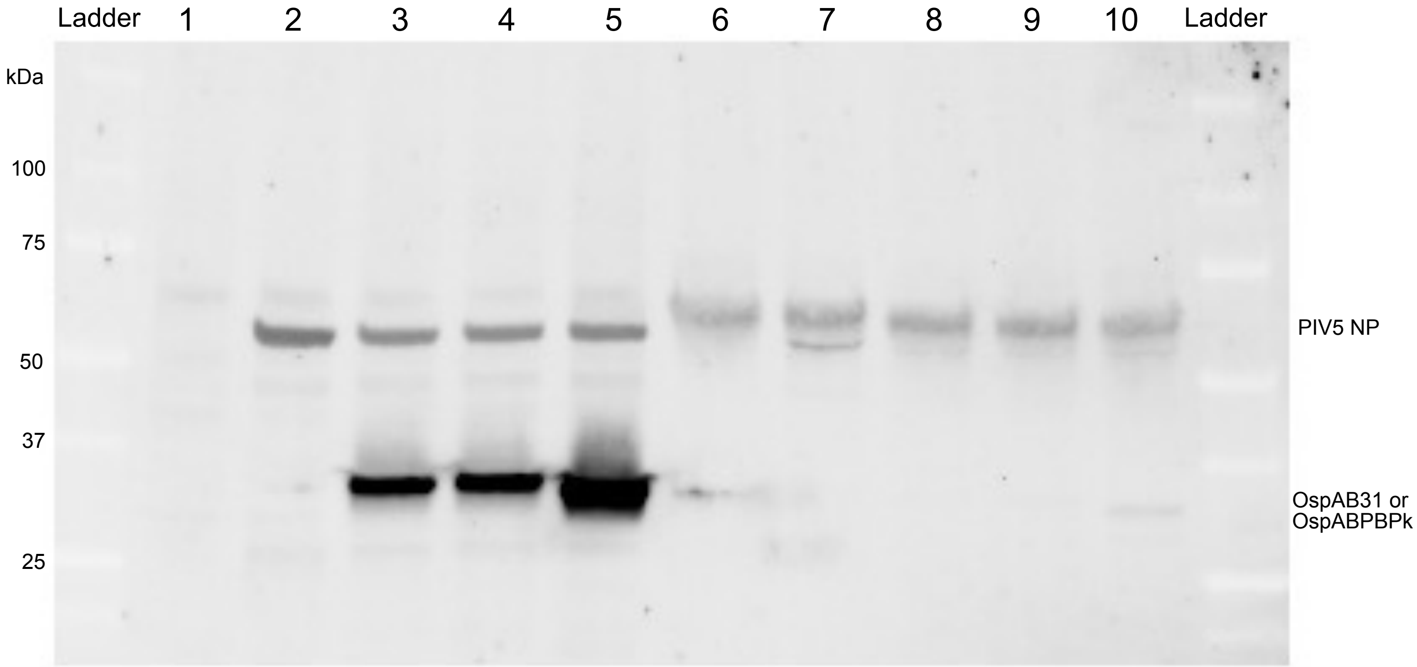

1. Mock cell lysate; 2. WT PIV5 cell lysate; 3. PIV5-OspAB31 cell lysate; 4. PIV5-OspABPBPk cell lysate; 5. PIV5-tPA-OspABPBPk cell lysate; 6.Mock supernatant; 7.WT PIV5 supernatant; 8.PIV5-OspAB31 supernatant; 9.PIV5-OspABPBPk supernatant; 10.PIV5-tPA-OspABPBPk sup.
